# Dynamic Modeling and Its Impact on Estimation Accuracy for GPS Navigation Filters

**DOI:** 10.3390/s25030972

**Published:** 2025-02-06

**Authors:** Dah-Jing Jwo, Ta-Shun Cho, Birhanu Ayalew Demssie

**Affiliations:** 1 Department of Communications, Navigation and Control Engineering, National Taiwan Ocean University, 2 Peining Road, Zhongzheng, Keelung 202301, Taiwan; 11257079@mail.ntou.edu.tw; 2 Department of Business Administration, Asia University, 500 Liufeng Road, Wufeng, Taichung 41354, Taiwan; cho2022@asia.edu.tw

**Keywords:** extended Kalman filter, global positioning system, modeling, divergence

## Abstract

This study addresses the divergence issues in GPS navigation systems caused by inaccuracies in dynamic modeling and explores solutions using the extended Kalman filter (EKF). Since algorithms such as the Kalman filter (KF) and EKF rely on assumed process models that often deviate from real-world conditions, their performance in real-time applications can degrade. This paper introduces fictitious process noise as an effective remedy to mitigate divergence, demonstrating its benefits through covariance estimation and tuning factors to enhance observability and controllability, particularly for continuous differential GPS (DGPS) access. The study evaluates several motion scenarios, including stationary receivers, straight-line trajectories with constant and varying speeds, and turning trajectories. The inclusion of process noise allows the EKF to adapt to changes in direction and speed without explicitly modeling turning or acceleration dynamics. To ensure robustness, the simulations incorporate a variety of scenarios to assess the statistical reliability and real-world performance of the EKF, ensuring the findings are statistically robust and widely applicable. Simulated receivers were used to evaluate the position (P), position–velocity (PV), and position–velocity–acceleration (PVA) models. The results from both the Ordinary Least-Squares (OLS) and EKF simulations show improved vehicle trajectory tracking and demonstrate the EKF’s potential for broader navigation system applications. This paper’s novel contribution lies in its thorough analysis of the divergence issues in GPS navigation filter designs due to dynamic modeling inaccuracies, providing a systematic approach to addressing these challenges and offering new insights to improve estimation accuracy.

## 1. Introduction

One of the most effective state estimators that yields a solution with the minimum mean square error (MMSE) is the Kalman filter (KF), which is used to analyze a sequence of noisy observations in order to estimate the state vector of a physical system [1,2,3,4,5,6]. The extended Kalman filter (EKF), a nonlinear variant of the KF, has been extensively applied in navigation and tracking domains, including radar tracking, GPS receiver position–velocity estimation, and integrated navigation systems [4,7,8,9]. Most GPS receivers utilize Kalman filters or least-squares estimation algorithms for position determination. Despite their widespread use, Kalman filters, including the EKF, present significant challenges, both conceptually and practically. These challenges stem from the filters’ reliance on accurate system models, proper noise covariance tuning, and iterative linearization in the case of the EKF, making them complex to implement and interpret. For those new to navigation and estimation, these complexities can lead to suboptimal performance or even filter divergence in real-world applications.

In Kalman filter designs, the modeling error-induced divergence problem is a major challenge. Divergence can be classified into two categories: (1) apparent divergence, where true estimation errors exceed theoretical predictions but remain bounded, and (2) true divergence, where estimation errors grow indefinitely. The performance of Kalman filters relies heavily on the process model, which is built on assumptions about the real-world dynamics. However, in most real-world situations, having a perfectly accurate model is often unrealistic. When the model is inaccurate, the filter’s real-time performance can deviate from its theoretical expectations. Additionally, both the KF and EKF assume that the stochastic errors in the dynamic process and measurement models have known a priori statistics. In practice, these assumptions are often violated, further limiting the filters’ effectiveness. Essentially, the process model is based on presumed real-world dynamics, but it is rare to have a model that is perfectly known. If the model is imprecise, the filter’s performance in real time will typically differ from theoretical predictions. Another challenge with using the KF or EKF in real-world scenarios is the assumption that the stochastic error statistics are known in advance. In many cases, the assumptions about these error statistics do not hold true, and the system model and noise characteristics are not fully accurate in the real world.

The adaptive Kalman filter (AKF) has been the subject of much research in an effort to overcome the drawbacks of the traditional KF and attain optimal filter performance [10,11,12,13,14,15,16]. This approach allows dynamic adjustment of filter parameters to improve accuracy. The AKF can be based on online motion estimation, incorporating both provided signal and noise statistics. Numerous studies have focused on improving covariance matrix estimation using an innovation-based approach, leading to the development of Innovation Adaptive Estimation (IAE) [12,13]. Xia et al. [14] also developed the adaptive fading Kalman filter (AFKF), which uses suboptimal fading factors to adjust the state estimation [14,15,16]. By increasing the expected covariance matrix through a factor matrix, the AFKF enhances the filter’s ability to respond to changes in system dynamics. The Multiple Model Adaptive Estimation (MMAE) strategy, proposed by Bar-Shalom and Blom, utilizes multiple state estimation filters tailored to specific models. The Interacting Multiple Model (IMM) method improves state estimation accuracy in dynamically varying systems by operating these filters in parallel and enabling information exchange at each time step [17]. Initially used in target tracking [18,19], the IMM approach has been extended to GPS navigation [20,21]. Unlike the IMM, which relies on model switching, IAE focuses on parametric adaptation, making it widely adopted in various navigation applications [22,23].

To address nonconvergence caused by unmodeled states, it is important to consider the system dynamics that the current model fails to capture. One effective approach is to introduce process noise into the Kalman filter’s system model, which helps prevent divergence. By increasing the process noise covariance, the filter becomes more robust to corrupted data. Process noise enables the filter to accommodate minor variations in direction or speed without requiring explicit modeling of vehicle acceleration or the turning rate. However, when state estimation errors begin to diverge, incorrect modeling can cause problems, such as the error covariance matrix incorrectly converging to zero or becoming ineffective. This study examines how dynamic modeling influences estimation accuracy in EKF-based GPS navigation filters, offering valuable insights that go beyond typical tutorial or example-based studies [24,25,26,27]. The results enhance our understanding of dynamic modeling in state estimation and provide practical solutions to improve estimation accuracy in real-world applications. The lessons learned from the KF and EKF can be applied to a variety of data processing tasks, especially those involving dynamic modeling.

The remainder of this paper is organized as follows. Section 2 offers a brief overview of state vector estimation for GPS navigation. Section 3 discusses the dynamic process model used to describe receiver motion. Section 4 presents an illustrative example and discusses the application of the GPS extended Kalman filter across various scenarios using several typical dynamic models. Finally, Section 5 concludes the paper.

## 2. State Vector Estimation for GPS Navigation

This section provides an overview of using the extended Kalman filter (EKF) to process GPS pseudorange observations for estimating the navigation state vector. Two common techniques for GPS navigation solutions are Ordinary Least Squares (OLS) and extended Kalman filtering. While both are commonly used, the GPS EKF is generally preferred because GPS pseudorange measurements exhibit a nonlinear relationship with the state variables. Despite sometimes being referred to as the GPS Kalman filter, the EKF tends to yield better results than OLS, primarily due to its ability to filter out high-frequency noise, which improves the accuracy of the estimates.

By considering the GPS pseudorange observables to the n satellites, which can be expressed as

(1) $$\rho_{i}=\sqrt{{\left(x_{i}-x\right)}^{2}+{\left(y_{i}-y\right)}^{2}+{\left(z_{i}-z\right)}^{2}}+ct_{b}+v_{\rho_{i}},\;i=1,\ldots ,n$$

where $\left(x_{i},y_{i},z_{i}\right)$ are the three-dimensional i-th satellite’s positions, (x,y,z) is the three dimensions’ user position, speed of light is c, the receiver clock offset from system time is denoted by $t_{b}$, and $v_{\rho_{i}}$ is the pseudorange noise.

### 2.1. The GPS Solutions Based on Least-Squares (OLS) Approach

One way to linearize Equation (1) is to extend Taylor’s series around the nominal (usually the estimated) user position, denoted as $\left({\hat{x}}_{n},{\hat{y}}_{n},{\hat{z}}_{n}\right)$, and neglecting the higher terms. Defining ${\hat{\rho }}_{i}$ as $\rho_{i}$ calculated at $\left({\hat{x}}_{n},{\hat{y}}_{n},{\hat{z}}_{n}\right)$, we obtain

(2) $$\Delta \rho_{i}=\rho_{i}-{\hat{\rho }}_{i}=\varepsilon_{i1}\Delta x+\varepsilon_{i2}\Delta y+\varepsilon_{i3}\Delta z+ct_{b}+v_{\rho_{i}}$$

where

(3) $$\varepsilon_{i1}=\frac{{\hat{x}}_{n}-x_{i}}{{\hat{r}}_{i}};\;\varepsilon_{i2}=\frac{{\hat{y}}_{n}-y_{i}}{{\hat{r}}_{i}};\;\varepsilon_{i3}=\frac{{\hat{z}}_{n}-z_{i}}{{\hat{r}}_{i}}$$

$${\hat{r}}_{i}=\sqrt{{\left({\hat{x}}_{n}-x_{i}\right)}^{2}+{\left({\hat{y}}_{n}-y_{i}\right)}^{2}+{\left({\hat{z}}_{n}-z_{i}\right)}^{2}}$$

The vector $(\varepsilon_{i1},\varepsilon_{i2},\varepsilon_{i3})\equiv E_{i}$, i=1,…,n, is the line-of-sight vector from the user to the satellites. We can represent Equation (2) in a matrix formulation

(4) $$\Delta \rho =\left[\begin{array}{c} \Delta \rho_{1}\\ \Delta \rho_{2}\\ \Delta \rho_{3}\\ \vdots \\ \Delta \rho_{n} \end{array}\right]=\left[\begin{array}{cccc} \varepsilon_{11} & \varepsilon_{12} & \varepsilon_{13} & 1\\ \varepsilon_{21} & \varepsilon_{22} & \varepsilon_{23} & 1\\ \varepsilon_{31} & \varepsilon_{32} & \varepsilon_{33} & 1\\ \vdots & \vdots & \vdots & \vdots \\ \varepsilon_{n1} & \varepsilon_{n2} & \varepsilon_{n3} & 1 \end{array}\right]\left[\begin{array}{c} \Delta x\\ \Delta y\\ \Delta z\\ ct_{b} \end{array}\right]+v_{\rho_{i}}$$

further, it can be written as

(5) z=Bx+v

n×4 is the dimension of matrix B for n≥4, where B is commonly known as the visibility matrix or geometry matrix. The solution to Equation (5) based on the weighted least-squares (WLS) approach is given by

(6) $$\hat{x}={\left(B^{T}WB\right)}^{-1}B^{T}Wz$$

If we let W be an identity matrix, the result becomes the OLS solution $\hat{x}={\left(B^{T}B\right)}^{-1}B^{T}z$, which is performed as a reference solution for comparison with the EKF approaches.

### 2.2. GPS Navigation Processor Using the Extended Kalman Filter

The dynamic process model in the continuous-time form is normally described by the ordinary differential equation and the measurement model is presented as an algebraic equation, as follows:

(7a) $$\mathrm{Process}\;\mathrm{model}:\;\dot{x}=Fx+Gu$$

(7b) Measurement model: z=Hx+v

where the vectors u(t) and v(t) are mutually independent white-noise sequences both with zero means:

(8) $$E[u(t)u^{T}(\tau )]=q\delta (t-\tau );\;E[v(t)v^{T}(\tau )]=r\delta (t-\tau );\;E[u(t)v^{T}(\tau )]=0$$

where E [⋅] is the expectation, the matrix transpose is denoted by superscript “T”, and δ(t) represents the Dirac delta function. Equations (7a) and (7b) in discrete-time equivalent form can be written as

(9a) $$x_{k+1}=\Phi_{k}x_{k}+w_{k}$$

(9b) $$z_{k}=H_{k}x_{k}+v_{k}$$

where $x_{k}\in {ℜ}^{n}\;$ is the systems state vector, $w_{k}\in {ℜ}^{n}$ is the process noise vector, $z_{k}\in {ℜ}^{m}$ is the measurement vector, $v_{k}\in {ℜ}^{m}$ is the measurement noise vector, $\Phi_{k}=e^{F\Delta t}$ is the state transition matrix, and Δt is the sampling interval. In Equation (9), both the vectors $w_{k}$ and $v_{k}$ are zero-mean Gaussian white-noise sequences having zero crosscorrelation with each other

(10) $$E[w_{i}w_{k}^{T}]=Q_{k}\delta_{ik};\;E[v_{i}v_{k}^{T}]=R_{k}\delta_{ik};\;E[w_{i}v_{k}^{T}]=0\;\mathrm{for}\;\mathrm{all}\;i\;\mathrm{and}\;k$$

where $Q_{k}$ is referred to as the process noise covariance matrix, $R_{k}$ is referred to as the measurement noise covariance matrix, and $\delta_{ik}$ is known as the Kronecker delta function. The following is a summary of the discrete-time KF algorithm.

- Steps in prediction/time update equations:

(11) $${\hat{x}}_{k+1}^{-}=\Phi_{k}{\hat{x}}_{k}$$

(12) $$P_{k+1}^{-}=\Phi_{k}P_{k}\Phi_{k}^{T}+Q_{k}$$

- Steps in correction/measurement update equations:
  (13) $$K_{k}=P_{k}^{-}H_{k}^{T}{\left[H_{k}P_{k}^{-}H_{k}^{T}+R_{k}\right]}^{-1}$$
  (14) $${\hat{x}}_{k}={\hat{x}}_{k}^{-}+K_{k}[z_{k}-H_{k}{\hat{x}}_{k}^{-}]$$
  (15) $$P_{k}=[I-K_{k}H_{k}]P_{k}^{-}$$
  where $P_{k}$ is the error covariance matrix defined by $E[(x_{k}-{\hat{x}}_{k}){\left(x_{k}-{\hat{x}}_{k}\right)}^{T}]$, ${\hat{x}}_{k}$ is the estimate of $x_{k}$, and the weighting matrix $K_{k}$ is commonly known as the Kalman gain matrix. Equation (14) illustrates how the Kalman filter equations use a measurement value in the a priori estimation to produce a corrected a posteriori estimation.

The nonlinear systems of the following nonlinear stochastic differential equations can be processed by the extended KF, which can be represented as follows:

(16a) $$\dot{x}=f(x,t)+u(t)$$

(16b) z=h(x,t)+v(t)

The EKF algorithm bears some similarities to the KF algorithm, with a few changes. First of all, the state update equation becomes

(17) $${\hat{x}}_{k}={\hat{x}}_{k}^{-}+K_{k}[z_{k}-{\hat{z}}_{k}^{-}]$$

where

$${\hat{x}}_{k}^{-}=f_{k-1}({\hat{x}}_{k-1});\;{\hat{z}}_{k}^{-}=h_{k}({\hat{x}}_{k}^{-})$$

The relationship between the system and measurement matrices yields the linear approximation equations.

$$\Phi_{k}\approx {\left.\frac{\partial f_{k}}{\partial x_{k}}\right|}_{x_{k}={\hat{x}}_{k}^{-}};\;H_{k}\approx {\left.\frac{\partial h_{k}}{\partial x_{k}}\right|}_{x_{k}={\hat{x}}_{k}^{-}}$$

**Figure 1:**
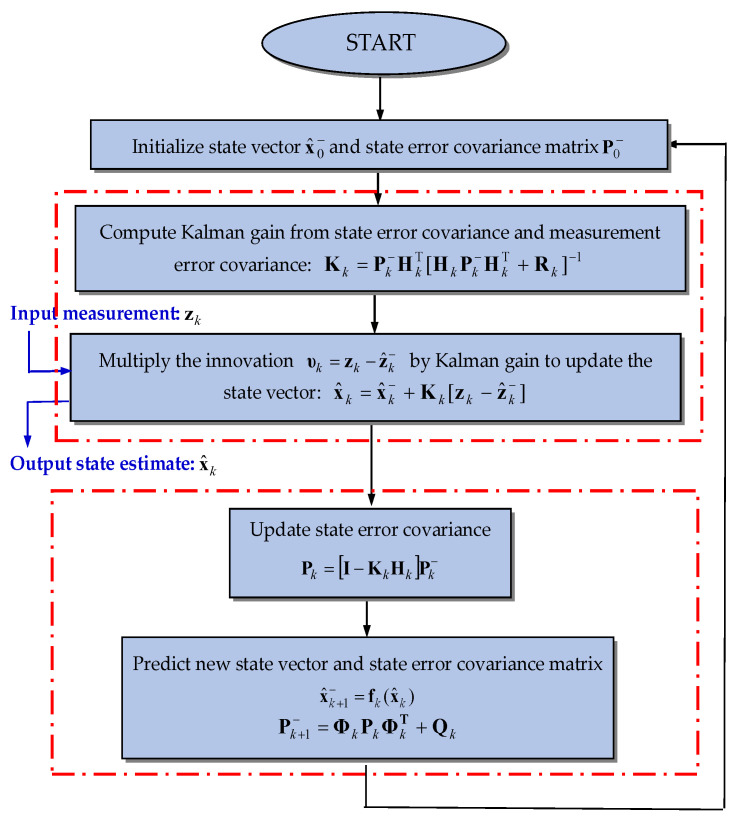
Flow chart for implementation of the extended Kalman filter.

**Figure 2:**
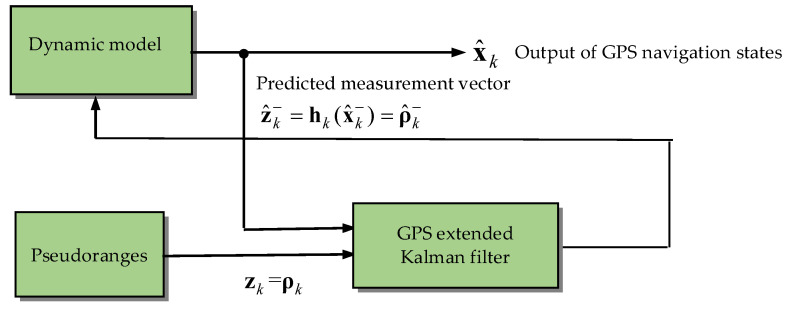
Configuration of the GPS extended Kalman filter.

The flow chart for the extended Kalman filter approach is shown in Figure 1. The Kalman filtering starts with the initial conditions ${\hat{x}}_{0}^{-}$ and $P_{0}^{-}$. While the new measurement $z_{k}$ is available, the state estimate ${\hat{x}}_{k}$ and the corresponding error covariance $P_{k}$ then proceed recursively infinite as time progresses. Figure 2 presents the configuration of the GPS extended Kalman filter. The GPS navigation filter employs the pseudorange observables as the measurement vector, which nonlinearly relates to the state vector. For a more thorough discussion, see Gelb [2] and Brown and Hwang [4].

To overcome the deficiency and furtherly achieve the filter optimality of the KF, ideally, it is useful to tune the filter for the entire range of motion types one expects it to filter. The IAE and fading factor approaches are popular as the noise-adaptive filter for tuning the noise covariance matrices. The IAE approach employs the innovative sequence $\upsilon_{k}=z_{k}-{\hat{z}}_{k}^{-}$, which is given from the incoming measurement $z_{k}$ and the state prediction ${\hat{x}}_{k}^{-}$ obtained in the previous step. The innovation reflects the discrepancy between the actual measurement $z_{k}$ and the predicted measurement ${\hat{z}}_{k}^{-}$. For the nonlinear measurement ${\hat{z}}_{k}^{-}=h_{k}({\hat{x}}_{k}^{-})$ while for the linear case, ${\hat{z}}_{k}^{-}=H_{k}{\hat{x}}_{k}^{-}$. By applying a factor matrix to the expected covariance matrix, fading Kalman filtering aims to purposefully raise the projected state vector’s variance: $P_{k+1}^{-}=\lambda_{k}\Phi_{k}P_{k}\Phi_{k}^{T}+Q_{k}$ where $\lambda_{k}=diag(\lambda_{1},\lambda_{2}\ldots ,\lambda_{m})$. The main difference between various fading memory algorithms is in the calculation of scale factor $\lambda_{k}$. When $\lambda_{i}=1$ (i=1,2,…,m), it eventually degrades to the standard KF.

The aim of this paper is not to design an adaptive estimator but to address divergence issues by refining covariance estimation and adding extra process noise to the system model. By introducing fictitious process noise, the study illustrates how the GPS EKF can be optimized, offering valuable insights and serving as a benchmark for future advanced filter designs. The focus is on enhancing estimation accuracy through adjustments to the covariance matrix and other sensor-related factors.

### 2.3. Connection Between Kalman Filter and Ordinary Least Squares

The KF’s update step can be thought of as the deterministic least-squares estimator. In order to improve and modify the state estimate, this method relies on incorporating fresh measurement data. The least-squares approach is mostly concerned with minimizing measurement residuals, or the difference between actual and projected measurements, as opposed to the KF, which is established by minimizing the solution’s mean-square error.

The $P_{k}$ and $K_{k}$ matrices based on the alternative form of the KF algorithm [4] can be represented as

$$P_{k}=\left[I-K_{k}H_{k}\right]P_{k}^{-}={\left[{\left(P_{k}^{-}\right)}^{-1}+H_{k}^{T}R_{k}^{-1}H_{k}\right]}^{-1}$$

and

$$K_{k}=P_{k}^{-}H_{k}^{T}{\left[H_{k}P_{k}^{-}H_{k}^{T}+R_{k}\right]}^{-1}=P_{k}H_{k}^{T}R_{k}^{-1}$$

respectively. For the deterministic least squares, no a priori knowledge of x is available, and ${\hat{x}}_{0}^{-}$ will be zero and its associated error covariance will be infinity: ${\hat{x}}_{0}^{-}\to 0$ and $P_{0}^{-}\to \infty$. Using the alternative form of the KF algorithm leads to $P_{0}^{-}={\left(\infty \right)}^{-1}+H_{0}^{T}R_{0}^{-1}H_{0}$ and the Kalman gain can then be written as $K_{0}=P_{0}^{-1}H_{0}^{T}R_{0}^{-1}=(H_{0}^{T}R_{0}^{-1}H_{0})H_{0}^{T}R_{0}^{-1}$ and thus the KF estimate of x at t=0 is given by

(18) $${\hat{x}}_{k}={\hat{x}}_{k}^{-}+K_{k}[z_{k}-{\hat{z}}_{k}^{-}]=K_{0}z_{0}=[(H_{0}^{T}R_{0}^{-1}H_{0})H_{k}^{T}R_{0}^{-1}]z_{0}$$

in the weighted least-squares problem with $R_{0}^{-1}$ playing the role of the weighting matrix W. The ability of the basic KF to automatically adjust the measurement weights gives it an edge over the deterministic least-squares solution. The theoretical relationship between the Kalman filter and ordinary least squares is discussed in reference [28,29].

### 2.4. Comparison Between Kalman Filter and Moving Average Filter

The moving average filter [30] and the Kalman filter are both used for smoothing data and reducing noise, but they differ significantly in their methods and applications. The moving average filter, or averaging filter, works by computing the average of a fixed number of recent input samples to produce the output. This simple, linear approach helps to reduce high-frequency noise and smooths the data. It assumes that the data consist of a mix of signal and high-frequency noise, but do not consider any underlying system dynamics or specific noise characteristics. Once the number of samples is determined, the moving average filter does not adapt to changing data or noise conditions.

In contrast, the Kalman filter is a more advanced, recursive algorithm designed to estimate the state of a system over time by combining noisy measurements with a model of the system’s dynamics. It generates optimal estimates by weighing the uncertainty in both the measurements and the system’s predictions. The Kalman filter assumes that the system can be described by state equations and that the noise follows a Gaussian distribution. It adapts continuously, updating estimates based on both prior knowledge and incoming data, which makes it well suited for dynamic and uncertain environments.

Mathematically, the moving average filter is relatively simple, as it requires just the calculation of an arithmetic mean for a set of input samples, with no need for additional computations or models. The Kalman filter, however, is more complex, using matrices to represent the system’s state and employing prediction and correction steps. It recursively updates estimates and requires a deep understanding of both the system’s dynamics and the measurement noise. Overall, the Kalman filter is more flexible and adaptable, making it better suited for systems where the conditions are constantly changing. The moving average filter, once set, remains static and does not adjust based on the nature of the data or the noise. For further details, refer to [30]. 

## 3. The Dynamic Process Model for Description of GPS Receiver Motion

Unless more sensors are available to reflect the actual dynamical behavior, a better model that is preferably nonlinear is challenging and unrealistic. There are problems with the accuracy of location estimates due to the modeling of vehicle motion and dynamic processes. The sensor presents a method to preserve the necessary model so that it does not diverge if there is not a better motion model to use or another way to handle the motion. Another problem is using other external motion sensors in conjunction with the Kalman filter motion model.

The **P** (position) model performs well when the vehicle or receiver is stationary since the presumed model accurately depicts the actual situation. The PV (position–velocity) motion model, on the other hand, is a viable option if the vehicle travels in a straight line at a steady pace. A simple motion model, such as the constant velocity model, can be used to successfully steer a vehicle that gradually changes its direction or speed over time. The assumption of constant velocity is shattered, though, when the vehicle begins to travel in different directions, and the PV motion model’s subpar performance results in filter divergence. For medium-dynamic applications like constant acceleration motion, more complex models like the PVA (position–velocity–acceleration) model are chosen.

(1) Dynamic model for the GPS receiver clock bias

Choosing the EKF as the GPS receiver’s navigation state estimator and representing the clock bias b and clock drift d, the differential equation for the receiver clock error is expressed as

(19) $$\dot{b}=d+u_{b}$$

$$\dot{d}=u_{d}$$

where $u_{b}\sim N(0,S_{f})$ and $u_{d}\sim N(0,S_{g})$ are white-noise sequences that are Gaussian-distributed and independent of each other.

(2) Dynamic model for a stationary receiver

This and the subsequent examples have linear dynamical equations. A five-state filter model, often known as the P (position) model, is the simplest motion model among others. The five-state filter is only suitable for very-low-dynamic applications, modeling three location states and two clock states. A stationary receiver can benefit from a five-state filter.

(3) Dynamic model for a low-dynamic receiver

The system with relatively stable velocity is designed to track an object that rotates slowly over a large distance at a constant speed. When evaluating a vehicle that slows down or turns quickly, it cannot perform as effectively. The constant velocity motion model (as random-walk process), also known as the PV (position–velocity) model, is used in an eight-state filter to assume that an object moves at a nearly constant speed. The eight-state filter can handle low-dynamic applications because it adds three velocity levels to the five-state filter.

(4) Dynamic model for a medium-dynamic receiver

The medium-dynamic trajectory, also known as the PVA (position–velocity–acceleration) model, is better accommodated by the 11-state filter, which extends the 8-state filter by 3 acceleration states. The 11-state filter is suitable for medium-dynamic applications. The PV and PVA models for GPS navigation are equivalent to the CV and CA models for radar tracking, respectively, except those two additional clock errors are involved in a GPS receiver. In Appendix A, the dynamic process and linearized measurement models of the GPS EKF and the associated noise parameters are summarized.

(5) Additional motion models that deal with time-varying velocity or a constant turn rate

There are several well-known motion models, like the constant turn, which assumes that the vehicle moves along a circular arc primarily at a constant speed. The TURN model (constant turn rate) and the Integrated Ornstein–Uhlenbeck (IOU) motion model are further possibilities that are considered. With factors that affect certain features of the velocity distribution, the IOU model seems promising because it can account for time-varying velocity.

## 4. Example Illustration and Discussions

This section presents several key cases and illustrative examples to assess the impact of dynamical model mismatches on performance. The examples will demonstrate how deviations in the model can lead to performance degradation. The standard dynamic models examined in the EKF include the P model for a stationary receiver, the PV model for a receiver with constant velocity, and the PVA model for a receiver with constant acceleration. We will compare the results obtained using the EKF and OLS with different assumed models.

To further illustrate the differences, supporting examples will show comparisons between OLS and the EKF. While other advanced nonlinear filters, such as the Unscented Kalman Filter (UKF) and Particle Filter (PF), are available, the EKF is selected for demonstration purposes, as it remains a widely applicable method. The linearization assumption holds true if the problem is sufficiently observable, ensuring that the differences between the estimated and actual trajectories remain relatively small.

The following examples cover a variety of dynamic behaviors and motion patterns to provide deeper insights into the performance of the EKF. These typical motion scenarios include the following: (1) stationary receiver, (2) straight-line trajectory with constant speed, (3) straight-line trajectory with varying speed, and (4) trajectory involving turning.

### 4.1. Scenario 1: Stationary GPS Receiver

Comparison of GPS navigation solutions based on the EKF approaches involving various dynamic models are provided, including the EKF using the three dynamic models: (1) P (ideal for a stationary receiver); (2) PV (ideal for a straight-line motion with constant velocity); and (3) PVA (ideal for a straight-line motion with constant acceleration), and the OLS as a reference solution.

The numerical experiment was conducted using a simulated receiver placed at the National Taiwan Ocean University (NTOU), located at 25.1492° N latitude, 121.7775° E longitude, and an altitude of 100 m. A coordinate transformation was applied to convert the geographic coordinates (latitude, longitude, altitude) into ECEF (Earth-Centered, Earth-Fixed) Cartesian coordinates. This results in the receiver’s location being expressed in meters within the WGS-84 ECEF coordinate system [7]. The origin of the local tangent ENU (East–North–Up) frame is defined at the point (0,0,0) meters.

The estimated level of measurement noise must be taken into account. Low-noise data can let the filter be modified to better monitor changes in the motion model. The impacts of the troposphere and ionosphere are thought to have been largely minimized by standard correction models utilizing the differential GPS (DGPS) mode; however, we believed that only thermal noise is involved on the pseudorange observables. The measurement noise variance $r_{\rho_{i}}$ value is assumed a priori known, which is 1$m^{2}$. Let each of the white-noise process noise be driven with the spectral amplitudes, referred to as the power spectral density (PSD): $S_{p}=0.05{\left(m/\sec\right)}^{2}/\left(rad/\sec\right)$. Furthermore, let the clock model spectral amplitudes be $S_{f}=0.4(10^{-18})\sec$ and $S_{g}=1.58(10^{-18})\sec^{-1}$. These spectral amplitudes can be used to generate the $Q_{k}$ matrix in Equation (A1).

**Figure 3:**
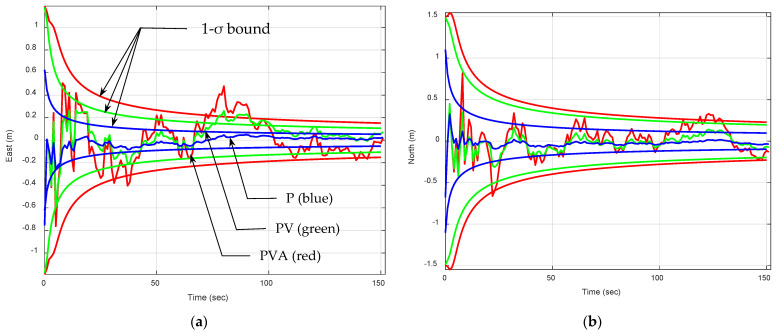
Position errors and the associated predicted 1-σ bound for a stationary receiver using the EKF, all with zero process noise covariance matrix $Q_{k}=0$. The blue, green, and red plots are results based on the P, PV, and PVA models, respectively: (**a**) east; (**b**) north.

Figure 3 provides the position errors and the associated predicted 1-σ bound. For all cases, the process covariance matrix $Q_{k}$ is set as zero matrices. For a stationary receiver, the P model aligns with the real-world environment. Its performance highlights the crucial role of selecting a model that closely aligns with the real motion characteristics of the navigation system being simulated. When the assumed model closely reflects the real world, $Q_{k}$ should be as small as possible. Even if the covariance matrix $Q_{k}=0$ is applied to all the various types of dynamic models, the PV and PVA induce more errors caused by modeling errors. Among the three dynamic models, the PVA model produces the poorest results due to inaccuracies in modeling the system dynamics. The lower Geometric Dilution of Precision (GDOP) suggests optimal use of satellite geometry, which improves positional accuracy under this configuration. Despite having a good GDOP, it exhibits significant variability in position errors, resulting from overfitting the dynamics (including unnecessary acceleration components). Estimation errors of the position and the corresponding one sigma confidence bound (1-σ bound) are presented for comparing the actual and theoretical estimation results. Theoretical results based on the 1-σ bounds match the actual estimation errors well. For clarity, Table 1 summarizes the comparison of the steady-state 1-σ error bound.

### 4.2. Scenario 2: Straight-Line Trajectory with Constant Speed

**Figure 4:**
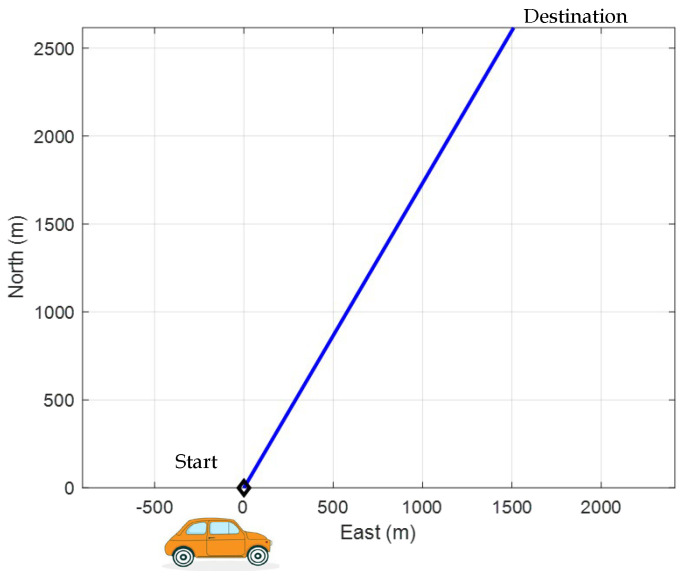
Test trajectory of the moving vehicle.

The next example considers a scenario involving a straight-line trajectory at a constant speed. The test trajectory is assumed to follow a straight path, as illustrated in Figure 4. A comparison of the solutions derived from the OLS and EKF algorithms is provided. The experiment was conducted with a simulated vehicle trajectory, starting at a position with coordinates 25.1492° N latitude, 121.7775° E longitude, and an altitude of 100 m.

(1) Comparison of results from EKF using three different dynamic models

**Figure 5:**
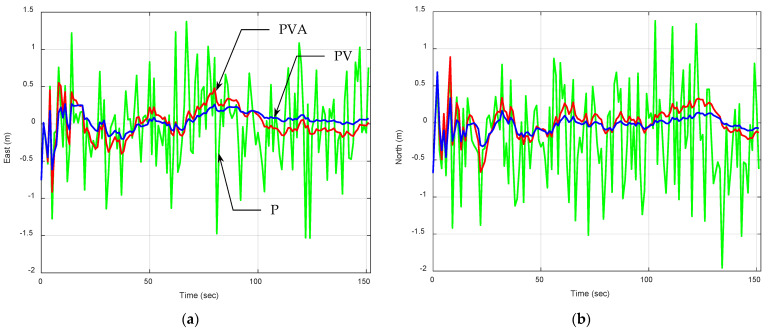
Position errors using the EKF for a vehicle traveling along a straight-line path at a constant speed. The green, blue, and red plots are the results based on P, PV, and PVA models, respectively. (PV and PVA models with zero process noise covariance matrix $Q_{k}=0$; P model with tuning factor ξ=1000): (**a**) east; (**b**) north.

**Figure 6:**
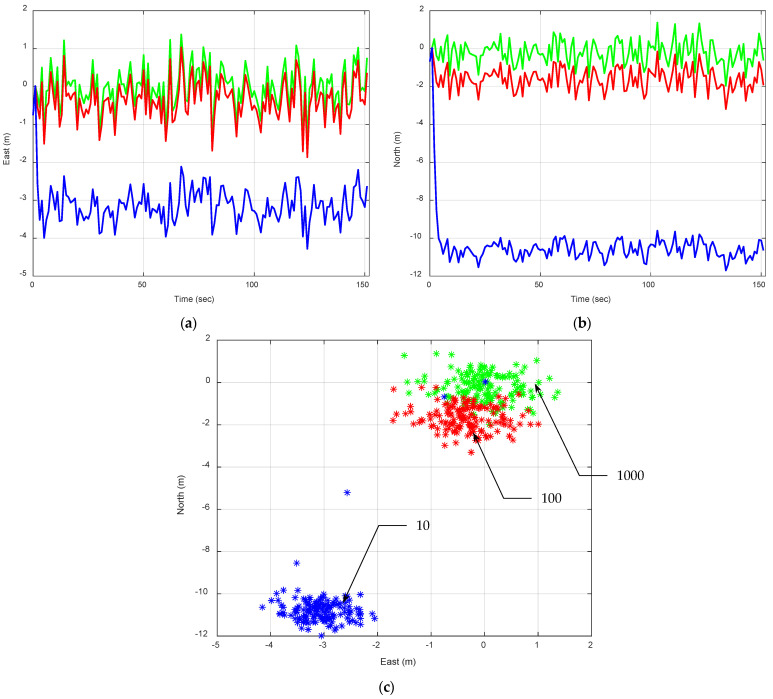
Position errors for a vehicle traveling along a straight-line path at a constant speed. The EKF using P model with increasing $Q_{k}$ values is presented. The green, red, and blue plots are results based on the tuning factor ξ=10, 100, and 1000, respectively: (**a**) east; (**b**) north; (**c**) 2D position errors.

The EKF algorithm incorporates the P, PV, and PVA dynamic models, with the PV model closely aligning with real-world dynamics. This makes it more robust for handling trajectory estimation, outperforming the P model (designed for stationary receivers) and the PVA model (intended for straight-line trajectories with constant acceleration). Figure 5 and Figure 6 illustrate the position errors using the EKF for a vehicle moving along a straight-line path at a constant speed. The parameter ξ is defined as the tuning factors of the process noise covariance matrix used to control the magnitude of fictitious process noise. Higher ξ values are better for scenarios with greater uncertainty or more complex dynamics, whereas lower ξ values are preferable when the motion model is very reliable or the environment is well controlled.

Model uncertainty can be compensated by increasing $Q_{k}$, which helps address mismatches in the dynamic process model. In Figure 5, the EKF using the PV and PVA models has a process noise covariance set to zero, i.e., $Q_{k}=0$. Meanwhile, when the P model is involved, the tuning factor ξ=1000 is utilized (namely, $Q_{k}=50$ ${\left(m/\sec\right)}^{2}/(rad/\sec)$ since the reference spectral amplitude is given by $S_{p}=0.05$${\left(m/\sec\right)}^{2}/(rad/\sec)$)). Table 2 summarizes the comparison of the error variance from the EKF using three different dynamic models for Scenario 2. The Kalman gain represents the filter’s confidence in the incoming measurements relative to the model’s predictions. When using the PV model with optimally tuned process and measurement noise, a well-balanced approach is achieved for tracking a vehicle moving at a constant speed along a straight path. The higher Kalman gain in the P and PVA models indicates a greater reliance on the incoming measurements, likely compensating for the limitations of these models in capturing motion dynamics or external variabilities. The error covariance matrix ($P_{k}$) reflects the accuracy of the state estimates. The significantly higher covariance in the P model suggests greater uncertainty in the position estimates, which aligns with its simpler dynamic modeling. In contrast, the PV and PVA models show lower covariance values, indicating tighter confidence intervals around their estimates. The PV model slightly outperforms the PVA3433117 model, likely due to its more specific adaptation to constant-speed conditions, without the added complexity of acceleration. Adjusting the process noise in the EKF allows the model to balance its trust in the process model against incoming measurements. A lower process noise reflects confidence in the model’s ability to predict future states, leading the filter to rely more on the model than on noisy measurements. Conversely, higher process noise indicates less trust in the model, making the filter more responsive to measurements, even if they are noisy.

Kalman filters account for measurement noise, helping the filter find the right balance between new measurements and the current motion model. There is an inherent trade-off with process noise: less process noise causes the filter to follow the motion model more closely, while ignoring abrupt deviations from the true trajectory. On the other hand, high process noise makes the filter more sensitive to noisy measurements, accepting larger local deviations from the model. Additionally, the filter tends to align more closely with the data when the measurement noise is low, which restricts how much the true trajectory can deviate from the motion model. When high measurement noise is specified, the filter favors the current motion model and responds more slowly to deviations, indicating the measurements are less reliable.

(2) Comparison of results based on EKF involving the P model with various $Q_{k}$ values

Figure 6 presents the EKF-based positioning errors using the P model, with an increase in $Q_{k}$. The results are obtained based on the tuning factor ξ=10, 100, and 1000, respectively, to control the magnitude of fictitious process noise, producing the spectral amplitudes $S_{p}=$ 0.5, 5, and 50${\left(m/\sec\right)}^{2}/\left(rad/\sec\right)$, respectively, since the reference spectral amplitude is set as $S_{p}=0.05{\left(m/\sec\right)}^{2}/\left(rad/\sec\right)$. The tuning factor for the process noise covariance in a Kalman filter setup should be chosen carefully, taking into account the expected system dynamics and the accuracy and reliability of the measurement data. Table 3 presents the comparison of the mean and variance of position errors using the P model with increasing $Q_{k}$ values for Scenario 2. It should be noted that the first five data points are considered transient and are excluded from the calculations of the mean and variance.

A moderate value is often the best compromise, striking a balance between being responsive to measurement updates and maintaining confidence in the model’s predictions. This is especially important in terms of its impact on bias error and error variance. By increasing ξ, we are signaling to the Kalman filter that there is more uncertainty in the movement model (position, in this case), which causes the filter to rely more on noisy measurements rather than the model’s predictions. Lower ξ values create a tighter connection to the model predictions, which is useful when the model is reliable and the system dynamics are well understood. On the other hand, higher ξ values make the filter more responsive to measurement data, which is beneficial in dynamic environments where model predictions are less certain. This leads to smaller mean errors, but at the expense of significantly higher error covariance, indicating lower precision.

The tuning factor effectively adjusts the amplitude of the fictitious process noise. It is important to appropriately adjust the spectral amplitudes of the process noise to match the vehicle’s dynamic characteristics. An increase in $Q_{k}$ to compensate for process model uncertainty is a remedy for nonconvergence. With an increasing $Q_{k}$, the bias error can be removed and the estimation errors approach zero mean at the expense of decreasing precision, namely increasing error variance. For the straight motion, a small $Q_{k}$ is appropriate. However, for pivots or more dynamics, a bigger $Q_{k}$ is used. By increasing the $Q_{k}$, the estimation precision declines, for which the outcome becomes increasingly noisy.

(3) Comparison of results based on EKF involving PV model with various $Q_{k}$

The EKF using the PV model with various values is presented for comparison with the OLS approach. The OLS method can be seen as having $Q_{k}$ set to infinity, which results in the poorest performance among the different approaches. When the model aligns well with the real world, the $Q_{k}$ value can be reduced to as small as possible to improve performance.

**Figure 7:**
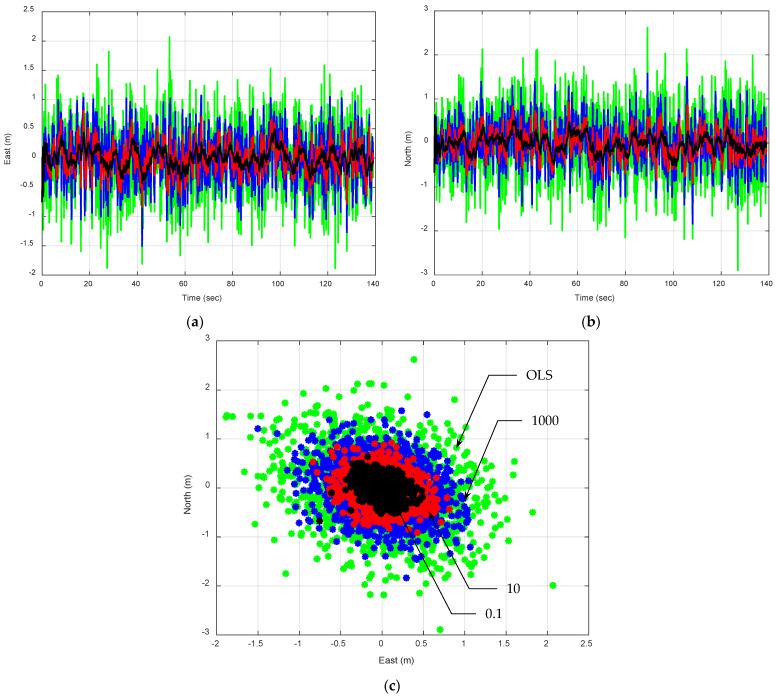
Position errors for a vehicle traveling along a straight-line path at a constant speed are analyzed using the EKF with the PV model and varying $Q_{k}$ values. The green, blue, red, and black plots are the OLS and the EKF with tuning factors ξ=0.1, 10, and 1000, respectively: (**a**) east; (**b**) north; (**c**) 2D position errors.

The estimation precision variation caused by increasing spectral amplitudes in the process noise is displayed in Figure 7. In this case, the assumed model in the Kalman filter matches the real world well. The tuning factors are set as ξ=0.1, 10, and 1000, respectively, and are used to control the magnitude of fictitious process noise in the process model, producing $S_{p}=$ 0.005, 0.5, and 50${\left(m/\sec\right)}^{2}/\left(rad/\sec\right)$, respectively. Table 4 presents a comparison of the mean and variance of position errors from the EKF using the PV model with increasing $Q_{k}$ values for Scenario 2. It can be observed that the error means are approximately zero in all cases, while the error variances increase as ξ increases, with the OLS results showing the largest variance. When process noise is high, the system is less sensitive to slight variations in timing measurements because it already accounts for large uncertainties in the model. However, it may result in increased error variance as the filter can become too reactive to measurement noise. Low process noise indicates high trust in the model’s ability to predict future states accurately. It results in the lowest error covariance, suggesting that the estimates are both precise and closely aligned with the model predictions. The noise characteristics of each sensor type must be accurately modeled in the EKF. This includes setting appropriate values for measurement noise covariance $R_{k}$ for each sensor type. The process noise covariance $Q_{k}$ also needs to be adjusted based on the confidence in the dynamic model relative to the sensor data quality. By integrating high-quality sensors and employing robust sensor fusion strategies, the dynamic model’s accuracy and reliability are significantly enhanced. This is particularly beneficial in environments where GPS signals are degraded or obstructed.

The primary determinant of the reference trajectory’s quality confidence is $Q_{k}$. For an extremely accurate model, the noise with zero procedure noise in the dynamic process covariance matrix (i.e., $Q_{k}=0$) will be adequate, resulting in a zero-estimation error. If the PSD (or equivalently, $Q_{k}$) is higher than what is required for convergence, the outcome becomes noisier without the mean error changing noticeably. Compared to the OLS approach, the basic Kalman filter has the advantage of automatically weighting the measurements appropriately depending on the geometry of the user satellites according to their contribution to the GDOP. As expected, the position estimation performance based on the EKF is noticeably improved when compared to the OLS solution. The OLS method, lacking the model-based predictions, shows worse performance compared to all EKF configurations. It solely depends on the raw measurements, which are not always reliable due to noise and other inaccuracies.

### 4.3. Scenario 3: Straight-Line Trajectory with Time-Varying Speed

**Figure 8:**
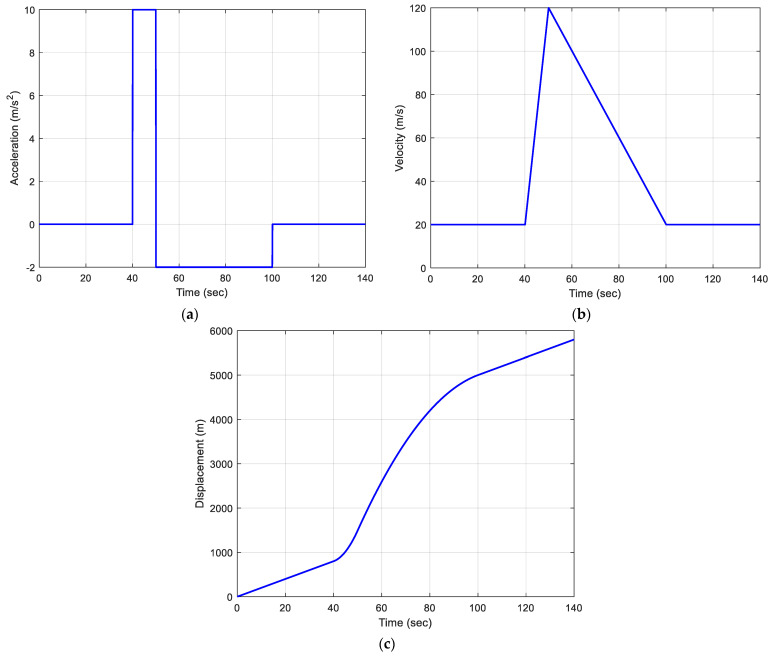
The acceleration, velocity, and displacement of a vehicle along the straight-line trajectory with time-varying speed: (**a**) acceleration; (**b**) velocity; (**c**) displacement.

Traditional methods often rely on fixed models, standard driving conditions, and constant vehicle speeds, which do not accurately capture real-world scenarios that involve sharp turns and variable speeds. This example tackles the navigation challenge of a car moving along a straight-line trajectory with time-varying speed. Along with the vehicle’s displacement, the corresponding acceleration and velocity are provided to give a deeper understanding of the vehicle’s dynamic behavior during the simulation, as shown in Figure 8. The example highlights three types of acceleration: large (40–50 s), small (50–100 s), and zero (constant velocity during the remaining two time intervals). The graphs showing acceleration, velocity, and displacement illustrate how dynamic changes unfold throughout the simulation.

**Figure 9:**
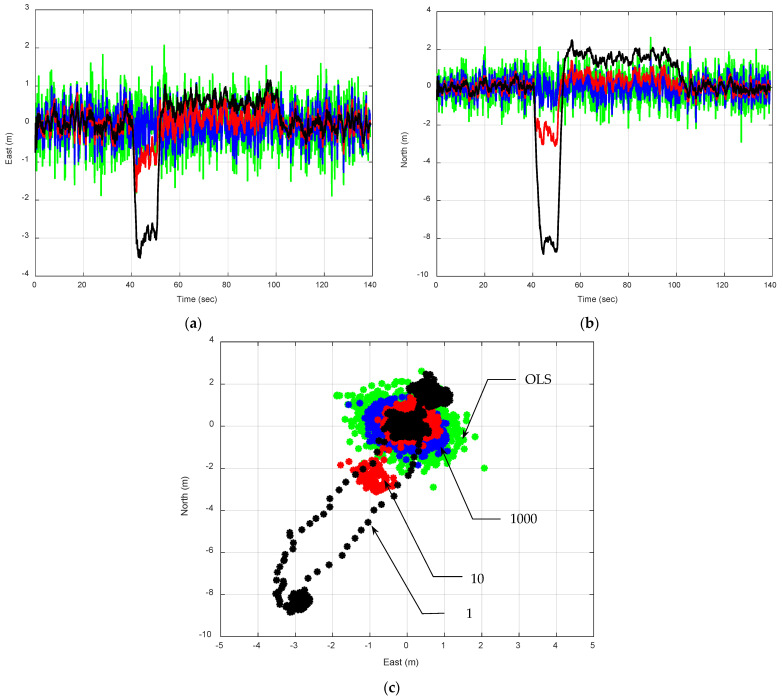
Position estimation errors for a vehicle moving along a straight-line trajectory at time-varying speed. The EKF using the PV model with increasing $Q_{k}$ is presented. The green, black, red, and blue plots are the OLS and the EKF with small (ξ=1) versus medium (ξ=10) and large (ξ=1000), respectively: (**a**) east; (**b**) north; and (**c**) 2D position errors.

The analysis focuses on a vehicle moving along a straight-line trajectory with time-varying speed, using different estimation models, particularly the PV model and EKF, to assess performance under varying levels of process noise. The PV model is employed with different amounts of process noise added to account for the small variations caused by speed changes. A comparison of EKF-based solutions involving the PV model (suitable for constant velocity motion) with various $Q_{k}$ is shown in Figure 9. The estimation error, however, is affected by the turning motion since it represents medium dynamics rather than low dynamics. Figure 9 shows the position estimation errors based on the OLS without dynamic adaptation and accounting for time-varying dynamics or noise explicitly. and the EKF with small (ξ=1), medium (ξ=10), and large (ξ=1000) spectral amplitudes, respectively, namely $S_{p}=$ 0.05, 0.5, and 50${\left(m/\sec\right)}^{2}/\left(rad/\sec\right)$, respectively. It can be observed that an offset exists between 50 and 100 s, particularly for the black line, which corresponds to the time period with time-varying speed. An adaptive process noise covariance scaling method marks a major advancement in Kalman filtering technology. It improves the filter’s capability to handle the inherent uncertainties and variabilities found in real-world situations, resulting in better accuracy, reliability, and robustness for navigation systems. This analysis provides a solid foundation for future research, especially for beginners, into adaptive filtering techniques that could autonomously adjust to changing environmental dynamics and measurement conditions.

### 4.4. Scenario 4: Trajectory Involving Turning

Acceleration can result from various dynamic scenarios, including turning. In this case, a car moves along a curved path at a constant speed. To account for the deviations caused by changes in steering and speed, a position–velocity (PV) model with added process noise is employed. The process noise helps capture the variations due to steering and speed changes during the turn, allowing the EKF to manage trajectory fluctuations while maintaining accuracy. Incorporating noise enables the filter to adapt to gradual motion changes that a fixed velocity model might overlook. The turn influences the estimation error because it introduces medium dynamics, in contrast to the low dynamics of straight-line motion.

**Figure 10:**
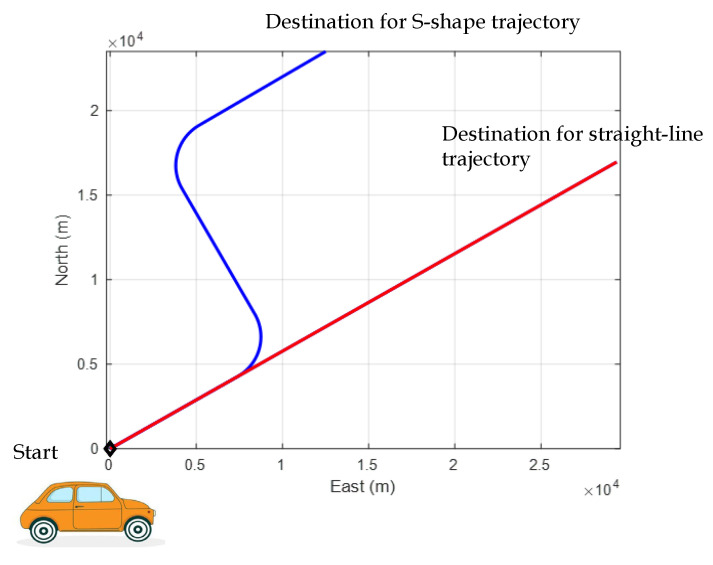
Two test trajectories, straight-line and S-shape trajectories, of the moving vehicle with constant speed.

(1) S-shape trajectory: This trajectory involving two 90-degree turns is chosen to evaluate the performance of the filter. The following is the simulated scenario. The actual trajectory is shown in blue in Figure 10. Two test trajectories, straight-line and S-shape trajectories, of the moving vehicle with constant speed are shown in Figure 10.

**Figure 11:**
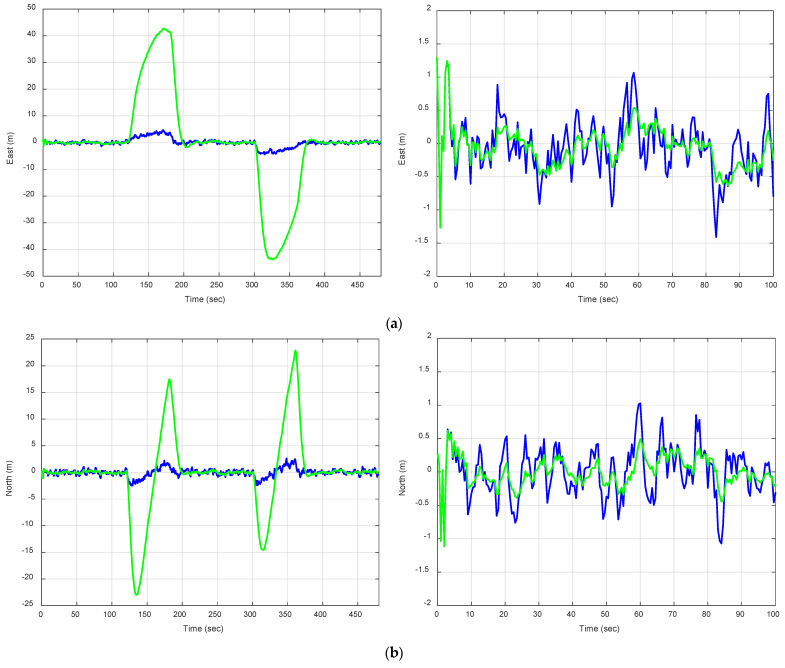
Position errors based on the PV model applied to two trajectories of a moving vehicle. The green and blue plots are the position errors for the S-shape trajectory with ξ=1 and ξ=0.01, respectively: (**a**) east error (**left**) and a closer look on the interval 0 to 100 s (**right**); (**b**) north error (**left**) and a closer look on the interval 0 to 100 s (**right**).

**Figure 12:**
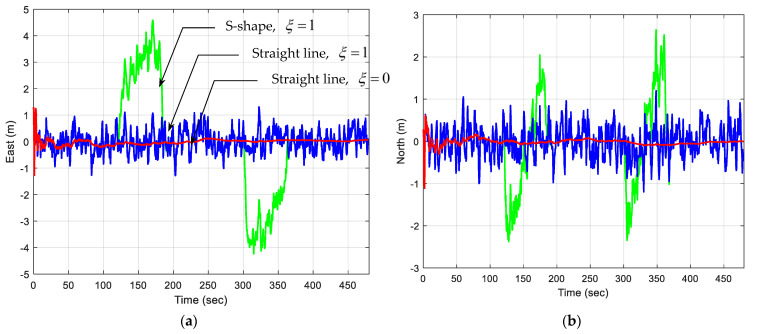
Position errors based on the PV model applied to two test trajectories of the moving vehicle. The green, blue, and red plots are S-shape trajectory with ξ=1 versus straight-line trajectory with ξ=1 and ξ=0, respectively: (**a**) east; (**b**) north.

A comparison of position estimation accuracies using the OLS approach versus the EKF with the PV model is presented to observe how the estimated EKF precision varies with increasing process noise. Figure 11 illustrates the position errors based on the PV model applied to the S-shaped trajectory of a moving vehicle. The green and blue plots are the position errors for the S-shape trajectory with ξ=1 and ξ=0.01, respectively. Figure 12 presents the position estimation accuracies based on the PV model applied to the S-shape trajectory as compared to the moving vehicle. The green, blue, and red plots are S-shape trajectories ξ=1 versus straight-line trajectories with ξ=1 and ξ=0, respectively. As the process noise increases, the EKF reduces position errors by better adjusting to abrupt directional changes. Quantitative metrics reveal that higher process noise increases Kalman gain and error covariance, enabling the EKF to effectively capture trajectory variations.

**Figure 13:**
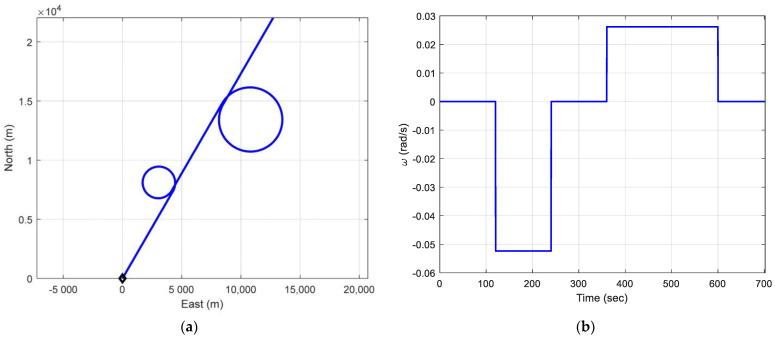
The motion along a straight line with two circular segments: (**a**) the trajectory; (**b**) angular velocity.

(2) Trajectory of straight line with two circles: The following is the simulated scenario. Within the simulation period, two circle trajectories are involved, with the genuine trajectory moving along a straight path. Two directions for the car’s movement were simulated: clockwise and counterclockwise. At the end of the simulation, the vehicle completes two circular motions. Figure 13 illustrates the motion along a straight line with two circular segments, displaying both the trajectory and angular velocity.

**Figure 14:**
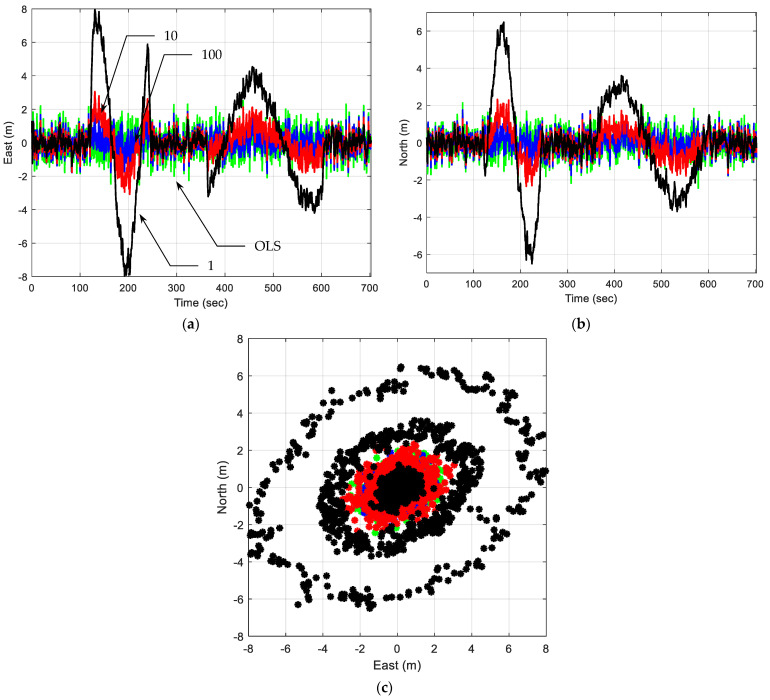
Position errors for a trajectory of straight line with two circles are shown. The green, blue, red, and black plots are the solutions using EKF with tuning factors ξ=1, 10, 100, and OLS, respectively: (**a**) east; (**b**) north; (**c**) 2D position errors.

Figure 14 presents the position errors of a trajectory of a straight line with two circles. The green, blue, red, and black plots are the solutions using the EKF with a tuning factor ξ=1, 10, 100, and OLS, respectively. Without incorporating appropriate process noise, an EKF using a constant velocity motion model will fit a straight line to the measurements. An EKF can accommodate gradual changes in direction or speed by giving more weight to recent observations compared to older ones. Although the terms “noise” and “uncertainty” imply deviations from the model, process noise allows for slight, predictable adjustments to a vehicle’s actual motion, which would otherwise require a more complex motion model.

With increasing process noise, the EKF reduces position errors by better adapting to abrupt directional changes. Quantitative metrics indicate that higher process noise increases Kalman gain and error covariance, allowing the EKF to effectively capture trajectory variations. For the circular trajectory, the EKF handles gradual directional changes effectively by weighting newer observations more heavily than older ones—a feature not available in the OLS approach. Without process noise, the EKF oversimplifies the motion as linear, leading to significant deviations. However, when appropriate process noise is introduced, the EKF can more closely align with actual circular trajectories, allowing for gradual, predictable deviations that would otherwise require a more detailed motion model.

In both scenarios, the PV model demonstrates robustness in handling medium-dynamic trajectories, with process noise playing a key role in balancing adaptability and accuracy. While OLS struggles with dynamic trajectories due to its static nature, the EKF’s recursive update mechanism and integration of process noise enable it to adapt effectively to both sharp and gradual changes, making it a superior choice for real-world applications such as autonomous vehicle navigation and traffic management. The ability to dynamically incorporate process noise makes the EKF highly effective in addressing medium-dynamic scenarios, offering a balance between precision and computational simplicity. These findings underline the importance of noise tuning for optimizing trajectory estimates and highlight the advantages of the EKF over static approaches like OLS, particularly in scenarios involving turning and curved motion.

## 5. Conclusions

This paper presents a comprehensive analysis of divergence issues in GPS navigation systems caused by inaccuracies in dynamic modeling and explores remedies for improving estimation accuracy using the EKF. It provides a detailed, section-wise explanation of how to address these issues, offering profound insights into the role of the EKF in mitigating filter divergence and improving estimation precision.

When estimating vehicle motion with an EKF, it is essential to account for unknown deviations from the assumed motion model. GPS receivers often exhibit departures from expected motion patterns, which can degrade filter performance. The EKF addresses this by introducing process noise to characterize the uncertainty or divergence between the vehicle’s actual motion and the presumed model. This process noise enables the filter to adapt to small deviations, enhancing its ability to handle real-world complexities in motion patterns, such as changes in direction or speed, without explicitly modeling the dynamics of acceleration or turning.

The study evaluates the estimation accuracy of the EKF in various dynamic scenarios involving the position (P), position–velocity (PV), and position–velocity–acceleration (PVA) models. Typical motion patterns considered include the following: (1) stationary receiver, (2) straight-line trajectory at constant speed, (3) straight-line trajectory with varying speed, and (4) trajectory involving turns. By manipulating the process noise covariance in the assumed system model, this paper demonstrates an effective solution for improving estimation accuracy and preventing filter divergence. This approach also provides valuable insights for enhancing dynamic modeling and encourages the exploration of advanced Kalman filter designs, such as adaptive and robust filters, for a variety of data processing applications. The addition of fictitious process noise allows the filter to respond to changes in direction or speed without requiring detailed calculations of turning or acceleration rates. By adjusting the process noise covariance, this study shows how to improve both the estimation accuracy and the overall robustness of the EKF, ultimately mitigating filter divergence.

In conclusion, this study offers significant insights into the divergence issues caused by dynamic modeling inaccuracies and provides practical solutions for improving GPS navigation filter accuracy. It provides a clear explanation of the EKF’s functionality, supported by illustrative examples, making it a valuable resource for those implementing GPS navigation filters, particularly when dealing with dynamic modeling. Additionally, the findings encourage further research into the use of the KF, EKF, and advanced filter designs for a wide range of data processing applications.

## Figures and Tables

**Table 1:** Comparison of steady-state 1-σ error bound (m) for Scenario 1.

	P	PV	PVA
East	0.0527	0.1062	0.1518
North	0.0975	0.1965	0.2281

**Table 2:** Comparison of the error variance (m^2^) from EKF using three different dynamic models for Scenario 2.

	P	PV	PVA
East	0.3035	0.0217	0.0516
North	0.4021	0.0170	0.0461

**Table 3:** Comparison of the mean and variance of position errors from EKF using the P model with increasing $Q_{k}$ values for Scenario 2.

ξ		10	100	1000
Mean (m)	East	−3.0672	−0.3267	−0.0084
	North	−10.8250	−1.6715	−0.2160
Variance (m^2^)	East	0.1638	0.2745	0.3064
	North	0.1685	0.3382	0.4088

**Table 4:** Comparison of the mean and variance of position errors from EKF using the PV model with increasing $Q_{k}$ values and the OLS method for Scenario 2.

ξ		0.1	10	1000	OLS
Mean (m)	East	−0.0019	−0.0021	−0.0020	−0.0021
	North	−0.0188	−0.0186	−0.0180	−0.0176
Variance (m^2^)	East	0.0248	0.0650	0.1729	0.3572
	North	0.0361	0.0990	0.2582	0.5660

## Data Availability

The data that support the findings of this study are available upon reasonable request from the authors.

## Appendix A. Dynamic Process and Linearized Measurement Models of the GPS Extended Kalman Filter and the Associated Noise Parameters

The state equations are representing the basic differential equations of motion. The process model can be governed by

$$\dot{x}=Fx+Gu$$

The dynamic process for a stationary receiver can be represented by the five-state P (position) model. In such cases, the GPS navigation filter involves three position states and two clock states, so that the state to be estimated is a 5×1 vector: $x={\left[\begin{array}{ccccc} x_{1} & x_{2} & x_{3} & x_{4} & x_{5} \end{array}\right]}^{T}$, where $x_{1}$, $x_{2}$, and $x_{3}$ represent the east, north, and vertical position; and $x_{4}$ and $x_{5}$ represent the receiver clock offset and drift errors, respectively. The state variables for the P model are driven by the white-noise processes: $u={\left[{\begin{array}{ccccc} u_{1} & u_{2} & u_{3} & u_{4} & u \end{array}}_{5}\right]}^{T}$. The dynamic process of the GPS receiver in a lower-dynamic environment can be represented by the eight-state PV (position–velocity) model. In such cases, the GPS navigation filter involves three positions in the east, north, and vertical components ($x_{1}$, $x_{3}$, $x_{5}$), three velocities in the east, north, and vertical components ($x_{2}$, $x_{4}$, $x_{6}$), and two clock states ($x_{7}$,$x_{8}$), so that the state to be estimated is an 8×1 vector: $x={\left[\begin{array}{cccccccc} x_{1} & x_{2} & x_{3} & x_{4} & x_{5} & x_{6} & x_{7} & x_{8} \end{array}\right]}^{T}$. The state variables for the PV model are driven by the white-noise processes: $u={\left[\begin{array}{cccccccc} 0 & u_{2} & 0 & u_{4} & 0 & u_{6} & u_{7} & u_{8} \end{array}\right]}^{T}$. The dynamic process of the GPS receiver in medium-dynamic applications can be represented by the 11-state PVA (position–velocity–acceleration) model. In such cases, the GPS navigation filter involves three position states ($x_{1}$, $x_{4}$, $x_{7}$), three velocity states ($x_{3}$, $x_{6}$, $x_{9}$), three acceleration states ($x_{2}$, $x_{5}$, $x_{8}$), and two clock states ($x_{10}$, $x_{11}$), so that the state to be estimated is an 11×1 vector: $x={\left[\begin{array}{ccccccccccc} x_{1} & x_{2} & x_{3} & x_{4} & x_{5} & x_{6} & x_{7} & x_{8} & x_{9} & x_{10} & x_{11} \end{array}\right]}^{T}$. The state variables are driven by the white-noise processes: $u={\left[\begin{array}{ccccccccccc} 0 & 0 & u_{3} & 0 & 0 & u_{6} & 0 & 0 & u_{9} & u_{10} & u_{11} \end{array}\right]}^{T}$.

Defining the following submatrices:

$$F_{P}=0;\;\;\;F_{PV}=\left[\begin{array}{cc} 0 & 1\\ 0 & 0 \end{array}\right];\;\;\;F_{PVA}=\left[\begin{array}{ccc} 0 & 1 & 0\\ 0 & 0 & 1\\ 0 & 0 & 0 \end{array}\right];\;\;\;F_{clk}=\left[\begin{array}{cc} 0 & 1\\ 0 & 0 \end{array}\right]$$

where the subscripts represent the submatrices to be utilized in the P, PV, PVA, and clock models, respectively. The F matrix can then be formed as follows:

$$F=\left[\begin{array}{cccc} F_{\mathrm{model}} & & & \\ & F_{\mathrm{model}} & & \\ & & F_{\mathrm{model}} & \\ & & & F_{clk} \end{array}\right],\;\mathrm{where}\;\mathrm{model}=P,\;\mathrm{PV},\;\mathrm{PVA}$$

Similarly, defining the following submatrices for the discrete-time model:

$$\Phi_{k,P}=1;\;\;\;\Phi_{k,PV}=\left[\begin{array}{cc} 1 & \Delta t\\ 0 & 1 \end{array}\right];\;\;\;\Phi_{k,PVA}=\left[\begin{array}{ccc} 1 & \Delta t & \Delta t^{2}/2\\ 0 & 1 & \Delta t\\ 0 & 0 & 1 \end{array}\right];\;\;\;\Phi_{k,clk}=\left[\begin{array}{cc} 1 & \Delta t\\ 0 & 1 \end{array}\right]$$

the state transition matrix can be found to be

$$\Phi_{k}=\left[\begin{array}{cccc} \Phi_{k,\mathrm{model}} & & & \\ & \Phi_{k,\mathrm{model}} & & \\ & & \Phi_{k,\mathrm{model}} & \\ & & & \Phi_{k,clk} \end{array}\right],\;\mathrm{where}\;\mathrm{model}=P,\;\mathrm{PV},\;\mathrm{PVA}$$

As for the covariance matrix, the following submatrices used in the P, PV, PVA, and clock models, respectively, are defined as follows:

$$Q_{k,P}=S_{p}\Delta t;\;\;\;Q_{k,PV}=S_{p}\left[\begin{array}{cc} \frac{\Delta t^{3}}{3} & \frac{\Delta t^{2}}{2}\\ \frac{\Delta t^{2}}{2} & \Delta t \end{array}\right];\;\;\;Q_{k,PVA}=S_{p}\left[\begin{array}{ccc} \frac{\Delta t^{6}}{20} & \frac{\Delta t^{4}}{8} & \frac{\Delta t^{3}}{6}\\ \frac{\Delta t^{4}}{8} & \frac{\Delta t^{3}}{3} & \frac{\Delta t^{2}}{2}\\ \frac{\Delta t^{3}}{6} & \frac{\Delta t^{2}}{2} & \Delta t \end{array}\right]$$

$$Q_{k,clk}=\left[\begin{array}{cc} S_{f}\Delta t+S_{g}\frac{\Delta t^{3}}{3} & S_{g}\frac{\Delta t^{2}}{2}\\ S_{g}\frac{\Delta t^{2}}{2} & S_{g}\Delta t \end{array}\right]$$

the process noise covariance matrices can be formed as follows:

(A1) $$Q_{k}=\left[\begin{array}{cccc} Q_{k,\mathrm{model}} & & & 0\\ & Q_{k,\mathrm{model}} & & \\ & & Q_{k,\mathrm{model}} & \\ 0 & & & Q_{k,clk} \end{array}\right],\;\mathrm{where}\;\mathrm{model}=P,\;\mathrm{PV},\;\mathrm{PVA}$$

where $S_{p}$, $S_{f}$, and $S_{g}$ are the spectral amplitudes.

If only the pseudorange observables are available, the linearized measurement equation based on n observables for the P, PV, and PVA models, respectively, are given by the following:

$$(P)\;\underset{Z_{k}}{\underset{︸}{\left[\begin{array}{c} \rho_{1}\\ \rho_{2}\\ \vdots \\ \rho_{n} \end{array}\right]-\left[\begin{array}{c} {\hat{\rho }}_{1}\\ {\hat{\rho }}_{2}\\ \vdots \\ {\hat{\rho }}_{n} \end{array}\right]}}=\underset{H_{k}}{\underset{︸}{\left[\begin{array}{ccccc} h_{x}^{\left(1\right)} & h_{y}^{\left(1\right)} & h_{z}^{\left(1\right)} & 1 & 0\\ h_{x}^{\left(2\right)} & h_{y}^{\left(2\right)} & h_{z}^{\left(2\right)} & 1 & 0\\ \vdots & \vdots & \vdots & \vdots & \vdots \\ h_{x}^{\left(n\right)} & h_{y}^{\left(n\right)} & h_{z}^{\left(n\right)} & 1 & 0 \end{array}\right]}}\left[\begin{array}{c} x_{1}\\ x_{2}\\ x_{3}\\ x_{4}\\ x_{5} \end{array}\right]+\left[\begin{array}{c} v_{\rho_{1}}\\ v_{\rho_{2}}\\ \vdots \\ v_{\rho_{n}} \end{array}\right]$$

$$(\mathrm{PV})\;\underset{Z_{k}}{\underset{︸}{\left[\begin{array}{c} \rho_{1}\\ \rho_{2}\\ \vdots \\ \rho_{n} \end{array}\right]-\left[\begin{array}{c} {\hat{\rho }}_{1}\\ {\hat{\rho }}_{2}\\ \vdots \\ {\hat{\rho }}_{n} \end{array}\right]}}=\underset{H_{k}}{\underset{︸}{\left[\begin{array}{cccccccc} h_{x}^{\left(1\right)} & 0 & h_{y}^{\left(1\right)} & 0 & h_{z}^{\left(1\right)} & 0 & 1 & 0\\ h_{x}^{\left(2\right)} & 0 & h_{y}^{\left(2\right)} & 0 & h_{z}^{\left(2\right)} & 0 & 1 & 0\\ \vdots & \vdots & \vdots & \vdots & \vdots & \vdots & \vdots & \vdots \\ h_{x}^{\left(n\right)} & 0 & h_{y}^{\left(n\right)} & 0 & h_{z}^{\left(n\right)} & 0 & 1 & 0 \end{array}\right]}}\left[\begin{array}{c} x_{1}\\ x_{2}\\ x_{3}\\ x_{4}\\ x_{5}\\ x_{6}\\ x_{7}\\ x_{8} \end{array}\right]+\left[\begin{array}{c} v_{\rho_{1}}\\ v_{\rho_{2}}\\ \vdots \\ v_{\rho_{n}} \end{array}\right]$$

$$(\mathrm{PVA})\;\underset{Z_{k}}{\underset{︸}{\left[\begin{array}{c} \rho_{1}\\ \rho_{2}\\ \vdots \\ \rho_{n} \end{array}\right]-\left[\begin{array}{c} {\hat{\rho }}_{1}\\ {\hat{\rho }}_{2}\\ \vdots \\ {\hat{\rho }}_{n} \end{array}\right]}}=\underset{H_{k}}{\underset{︸}{\left[\begin{array}{ccccccccccc} h_{x}^{\left(1\right)} & 0 & 0 & h_{y}^{\left(1\right)} & 0 & 0 & h_{z}^{\left(1\right)} & 0 & 0 & 1 & 0\\ h_{x}^{\left(2\right)} & 0 & 0 & h_{y}^{\left(2\right)} & 0 & 0 & h_{z}^{\left(2\right)} & 0 & 0 & 1 & 0\\ \vdots & \vdots & \vdots & \vdots & \vdots & \vdots & \vdots & \vdots & \vdots & \vdots & \vdots \\ h_{x}^{\left(n\right)} & 0 & 0 & h_{y}^{\left(n\right)} & 0 & 0 & h_{z}^{\left(n\right)} & 0 & 0 & 1 & 0 \end{array}\right]}}\left[\begin{array}{c} x_{1}\\ x_{2}\\ x_{3}\\ x_{4}\\ x_{5}\\ x_{6}\\ x_{7}\\ x_{8}\\ x_{9}\\ x_{10}\\ x_{11} \end{array}\right]+\left[\begin{array}{c} v_{\rho_{1}}\\ v_{\rho_{2}}\\ \vdots \\ v_{\rho_{n}} \end{array}\right]$$

where $v_{\rho_{1}}$ are additive white-noise measurements. The elements of the measurement model $H_{k}=\partial h_{k}/\partial x_{k}$ involving the direction cosine elements are the partial derivatives of the predicted measurements with respect to each state, which is an (n×5), (n×8), or (n×11) matrix, respectively. Assuming measurement errors among satellites are uncorrelated, we have the measurement noise covariance matrix:

(A2) $$R_{k}=\left[\begin{array}{cccc} r_{\rho_{1}} & & & 0\\ & r_{\rho_{2}} & & \\ & & \ddots & \\ 0 & & & r_{\rho_{n}} \end{array}\right]$$
